# All-cause and cause-specific hospitalization rates among temporary and permanent residents living in Canada: A linkage study

**DOI:** 10.17269/s41997-025-00996-9

**Published:** 2025-03-18

**Authors:** Jenney Meng Han Wang, Edward Ng, Dafna Kohen, Rachel Viau, Claudia Rank, Anne Grundy

**Affiliations:** 1 Canadian Field Epidemiology Program, Public Health Agency of Canada, Ottawa, ON Canada; 2 Migration Health Branch, Immigration, Refugees and Citizenship Canada, Ottawa, ON Canada; 3 Health Analysis Division, Statistics Canada, Ottawa, ON Canada; 4 Department of Public Health Sciences, Queen’s University, Kingston, ON Canada

**Keywords:** Migrant, Hospitalization rate, Hospital discharge, Temporary resident, Permanent resident, Immigrant status transition, Migrant, Taux d’hospitalisation, Sortie de l’hôpital, Résident temporaire, Résident permanent, Changement de statut d’immigration

## Abstract

**Objective:**

Increased understanding of migrant health outcomes is important for health policy and planning and to support continuity of care for Canadian newcomers. The objective of this study is to expand on previous migrant health research by examining age-standardized hospitalization rates (ASHR) among temporary residents (TRs) and permanent residents (PRs) living in Canada from 2014 to 2018.

**Methods:**

Hospitalization outcomes were obtained by linking administrative health databases to the Longitudinal Immigration Database. TRs and PRs were characterized by covariates including age, sex, migration category, and immigration status transition. All-cause and select cause-specific ASHRs were calculated, including hospitalizations for cancer, injury, and mental and health conditions.

**Results:**

All-cause ASHRs were lower among TRs than among PRs, with variations observed within specific migration categories. Among TRs, the ASHR was highest for temporary foreign workers. Workers had the highest ASHR for cancer and injury, while asylum claimants had the highest ASHR for mental health conditions. Among PRs, ASHRs were highest for refugees overall and for all specific causes examined. People who transitioned from TR to PR status had higher ASHRs overall compared to those who did not.

**Conclusion:**

Observed ASHR differences between TRs and PRs, and among those with immigration status transitions and within specific migration categories, may be related to selection criteria by migrant stream, differential access to healthcare resources, preventive health behaviours, and different exposures influencing health needs. Additional research on characteristics associated with migrant health can inform post-arrival health planning and continuity of care.

**Supplementary Information:**

The online version contains supplementary material available at 10.17269/s41997-025-00996-9.

## Résumé

**Objectif:**

Il est essentiel d’avoir une meilleure compréhension des résultats relatifs à la santé des migrants dans le cadre de l’élaboration de plans et de politiques en matière de santé afin d’assurer la continuité des soins pour les nouveaux arrivants au Canada. L’objectif de cette étude est d’approfondir les recherches antérieures sur la santé des migrants en examinant les taux d’hospitalisation normalisés selon l’âge (THNA) parmi les résidents temporaires (RT) et les résidents permanents (RP) qui vivaient au Canada entre 2014 et 2018.

**Méthode:**

Les résultats en matière d’hospitalisation ont été obtenus en associant les bases de données administratives sur la santé à la Base de données longitudinales sur les immigrants. Les RT et les RP ont été caractérisés par des covariables qui comprenaient l’âge, le sexe, la catégorie de migration et le changement de statut d’immigration. Les THNA pour toutes les causes confondues et pour certaines causes précises ont été calculées, y compris les hospitalisations liées à un cancer, à des blessures, à des problèmes de santé mentale et à l’état de santé.

**Résultats:**

Les THNA pour toutes les causes confondues étaient plus faibles chez les RT que chez les RP; des variations au sein de catégories de migration précises ont été observées. Chez les RT, le THNA était le plus élevé parmi les travailleurs étrangers temporaires. Les travailleurs avaient le THNA le plus élevé pour le cancer et les blessures, tandis que les demandeurs d’asile avaient le THNA le plus élevé pour les problèmes de santé mentale. Chez les RP, les THNA étaient les plus élevés parmi les réfugiés en général et pour toutes les causes précises qui ont été examinées. Les personnes qui sont passées du statut de RT à celui de RP avaient généralement des THNA plus élevés que celles qui n’ont pas fait la transition.

**Conclusion:**

Les différences observées en matière de THNA chez les RT et les RP, ainsi que parmi les personnes ayant changé de statut d’immigration et les personnes appartenant à des catégories de migration précises, peuvent être liées à des critères de sélection axés sur le volet de migration, sur les différences d’accès aux ressources de soins de santé, sur des comportements préventifs en matière de santé et sur différentes expositions qui influencent les besoins liés à la santé. Des recherches supplémentaires sur les caractéristiques liées à la santé des migrants peuvent contribuer à l’élaboration de plans en matière de santé après l’arrivée et à assurer la continuité des soins.

## Introduction

In 2021, Canada welcomed nearly 406,000 permanent residents (PRs) and almost 1.5 million temporary residents (TRs), and over 191,000 temporary workers and students transitioned from TR to PR status (Immigration, Refugees and Citizenship Canada, 2022). Canada plans to welcome immigrants at a rate of 1% of the Canadian population annually and by 2036, immigrants are projected to represent close to 30% of the Canadian population (Immigration, Refugees and Citizenship Canada, 2023). Continued migrant health research is important to inform health policy and planning, to support continuity of care after arrival, and to plan for health system implications of migrant populations in Canada (Gushulak et al., 2011).

Although many previous studies have examined hospitalization events and health status among PRs relative to Canadian-born populations (Grundy et al., 2023; Ng et al., 2021, 2016a, 2016b; Wallar & Rosella, 2020), few have examined health status differences within and between PRs and TRs (Lu et al., 2015; Wiedmeyer et al., 2023). As compared with PRs, healthcare coverage and eligibility vary between TR migrant groups and potentially by Canadian province/territory, which may result in different health outcomes after arrival.

The objective of this descriptive study is to expand on previous research (Grundy et al., 2023; Ng et al., 2021, 2016a, 2016b) by comparing all-cause and cause-specific hospitalization rates of different migrant groups, stratified by age, sex, and other selected characteristics, with a specific focus on the potential impact of migration status changes on migrant hospitalization rates.

## Methods

### Data linkage

Statistics Canada’s Longitudinal Immigration Statistical Environment (LISE) allows for the examination of health-related and other outcomes for immigrants and non-immigrants by providing linkage keys between the Longitudinal Immigration Database (IMDB), the Longitudinal Administrative Databank, and other data sources, including several maintained and provided by the Canadian Institute for Health Information (CIHI) (Statistics Canada, 2022).

The IMDB contains unduplicated immigrant records derived from the Immigration Landing Files for PRs and permits for TRs from Immigration, Refugees and Citizenship Canada (IRCC) with tax files from the Canada Revenue Agency. Since the 2018 release, the IMDB contains administrative information for all individuals landing in Canada since 1952, with data provided monthly to Statistics Canada by IRCC, and TR permits since 1980. The version of the LISE used for the current study included the 2019 IMDB for linkage to other files.

The Discharge Abstract Database (DAD), provided annually to Statistics Canada by CIHI, contains demographic, administrative, and clinical data for all acute-care and some psychiatric, chronic rehabilitation, and day-surgery discharges for all provinces and territories, excluding Quebec.

The Ontario Mental Health Reporting System (OMHRS), also provided annually to Statistics Canada by CIHI, includes records for all individuals admitted to designated inpatient mental health beds in general and specialty facilities in Ontario since fiscal year 2006/2007, including information at the assessment level about patients’ mental and physical health, social supports, and service use.

### Study cohorts

The study cohort was derived for PRs and TRs from the 2019 IMDB in LISE. The TR cohort included individuals with a valid TR permit on January 1, 2014, and included workers, students, and asylum claimants. The PR comparison group included individuals who had become PRs in Canada between January 1, 2000, and December 31, 2013, and included individuals in the economic, family, and refugee categories. Both TR and PR cohorts were followed from January 1, 2014, to December 31, 2018. TRs and PRs who landed in Quebec were excluded since hospitalization data are not available.

To examine the impact of immigration status transitions on hospitalization rates, TRs were further divided into two groups: TRs who remained TRs throughout the follow-up period (TRs only) and TRs who became PRs during the follow-up period (TR-PR). The PR comparison cohort was also divided into two groups: PRs who previously had TR status before landing as PRs (PR-TR) and those who had no previous TR permit status (PR only). Consequently, four subgroups were followed in this study.

### Acute-care all-cause and cause-specific hospitalization

**Fig. 1:**
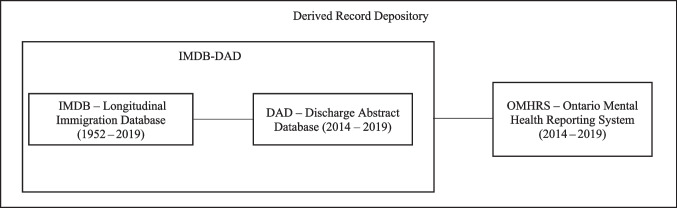
Temporary and permanent resident cohort based on the 2014–2018 IMDB-DAD-OMHRS linked database

Hospital discharges from the DAD or OMHRS between January 1, 2014, and December 31, 2018, were examined (Fig. 1). Hospital discharges were classified using the diagnosis or medical condition most responsible for the individual’s hospital stay, coded using the International Classification of Diseases and Related Health Problems, Canada, Tenth Revision (ICD-10-CA) and the Diagnostic and Statistical Manual of Mental Disorders, Fifth Edition (DSM-V) in the DAD and OMHRS, respectively (Supplementary Table 1). CIHI documentation was used to ensure consistency in diagnostic classifications between the two coding sources (Canadian Institute for Health Information, 2021). Specific hospitalization causes of interest for migrant populations were examined, including cancer, injury, mental health conditions (including self-inflicted injuries), vaccine-preventable diseases (VPDs), tuberculosis (TB), and other infectious diseases. The top three diseases for each specific cause were ranked by distribution for TRs and PRs.

### Covariates

Age and sex were determined for both TRs and PRs based on the IMDB. Age was calculated as the difference between the start of the follow-up period and the birth year recorded in the IMDB and grouped as 0–17, 18–39, 40–64, and 65 and older. In addition, age at arrival was recorded as the age at the start of the first permit for TRs and the age at landing for PRs.

Migrant characteristics such as age at and time since arrival in Canada, world region of origin, and admission category (for PRs) or permit category (for TRs) were obtained from the IMDB. Countries of origin, based on country of birth, were grouped into ten world regions: the United States, Caribbean and Central and South America, Western Europe, Eastern Europe, sub-Saharan Africa, Southwest Asia and North Africa, South Asia, Southeast Asia, East Asia, and Oceania. PR admission categories were classified as economic, family, refugees, and others (Minister of Justice, 2001). TRs were classified into three categories: temporary foreign workers, students, and asylum claimants.

### Statistical methods

Descriptive statistics were used to characterize TRs and PRs in the study. Crude and age-standardized hospitalization rates (ASHRs) per 10,000 life years were calculated overall and by the selected hospitalization causes for the follow-up period. To account for potential loss to follow-up due to death or emigration, rate derivation and time at risk was adjusted for individuals who died during follow-up for PRs, or for the duration of the last valid consecutive permit for TRs. In addition, since IMDB includes everyone who landed since 1952, the absence of tax filing for those aged 18 and above is used as a proxy for emigration. Specifically, PRs who arrived more than 2 years prior to January 1, 2014, but did not file taxes for the latest 2 years were assumed not to be in Canada, and were excluded from the analysis. For TRs, the same rates were calculated with follow-up based on when their last consecutive TR permit expired or December 31, 2018, whichever date came first. The ASHRs were further stratified by age at and time since arrival in Canada, sex, world region of origin, and migration categories.

## Results

The TR and PR cohorts are described in Table 1. Subgroup analysis showed that 45% of TRs became PRs during the follow-up period (i.e., TR-PRs). Among PRs, most (79%) originally came to Canada as PRs and had never been TRs (PRs only). The TR cohort was younger compared to the PR cohort: over half (64%) of TRs were aged 18 to 39 years, compared to 41% of PRs, with median ages of 26 and 30, respectively. More than half (63%) of TRs had been in Canada for less than 3 years, while close to half (49%) of PRs had been in Canada for 8 or more years. Approximately a third of TRs were classified as asylum claimants (36%), workers (34%), and students (30%), while the majority of PRs were from the economic class (59%), compared to the family (29%) and refugee (11%) classes. The top source regions for both TRs and PRs were similar, with more than a third (38%) of TRs and nearly half (46%) of PRs originating from South and East Asia.

**Table 1:** Descriptive characteristics of the TR and PR cohort overall and by covariates

	Temporary resident (TR) cohort			Permanent resident (PR) cohort		
	All TRs (*n* = 871,955)	Group A: TR only (*n* = 481,975)	Group B: TR who became PR (*n* = 389,980)	All PRs (*n* = 2,834,695)	Group A: PR only (*n* = 2,232,645)	Group B: PR who were previously TR (*n* = 602,050)
Overall		55%	45%		79%	21%
Age groups						
0–17	5%	7%	3%	14%	16%	4%
18–39	64%	64%	64%	41%	39%	52%
40–64	27%	26%	29%	38%	38%	41%
65 +	3%	3%	3%	7%	8%	3%
Age at arrival						
0–17	12%	13%	10%	25%	29%	12%
18–39	73%	71%	76%	51%	46%	66%
40–64	14%	15%	13%	21%	21%	21%
65 +	1%	1%	1%	3%	4%	1%
Time since arrival in Canada						
Less than 3 years	63%	61%	65%	14%	13%	18%
3–7 years	13%	15%	9%	36%	35%	42%
8 or more years	25%	24%	26%	49%	52%	40%
Sex						
Male	57%	61%	53%	48%	48%	50%
Female	43%	39%	47%	52%	52%	50%
Immigrant admission categories						
Temporary residents						
Worker	34%	28%	42%			
Student	30%	35%	24%			
Asylum claimant	36%	37%	34%			
Permanent residents						
Economic				59%	61%	51%
Family				29%	31%	22%
Refugees				11%	7%	22%
Source world region						
USA	6%	8%	2%	3%	3%	5%
Caribbean/Central and South America	1%	17%	9%	8%	6%	14%
Western Europe	7%	8%	6%	5%	4%	9%
Eastern Europe	9%	10%	8%	8%	8%	8%
Sub-Saharan Africa	7%	6%	9%	6%	5%	9%
Southwest Asia with North Africa	8%	7%	9%	12%	13%	7%
South Asia	16%	9%	24%	25%	28%	13%
Southeast Asia	9%	3%	15%	11%	11%	13%
East Asia	22%	27%	17%	21%	21%	19%
Oceania	2%	3%	1%	1%	0%	2%

Source: 2014–2018 IMDB-DAD-OMHRS linked database, linked through the Longitudinal Immigration Statistical Environment (LISE), a secure linkage environment containing anonymous linkage keys of a subset of databases

### All-cause ASHR

Table 2 shows the overall all-cause crude and ASHRs of TRs and PRs by various characteristics. Overall, the ASHR among TRs (191 per 10,000) was almost half that of the PR cohort (376 per 10,000). Among TRs, the ASHR was highest among workers, followed by asylum claimants and students. Among PRs, the ASHR was highest among refugees, followed by the family and economic class. The all-cause crude and ASHRs were much higher for females than for males among TRs and PRs. Among TRs, those from Southeast Asia and Western Europe had the highest ASHRs, while PRs from sub-Saharan Africa and the Caribbean and Central and South America had the highest ASHRs.

**Table 2:** All-cause crude and age-standardized hospitalization rates (ASHR) for temporary and permanent residents, by selected characteristics (per 10,000 life years)

	Temporary residents (TRs)			Permanent residents (PRs)		
	Crude rate	ASHR	95% confidence interval	Crude rate	ASHR	95% confidence interval
Overall	235.5	191.3	188.1–194.5	375.0	375.8	374.3–377.2
Age at arrival						
0–17	61.5			203.3		
18–39	263.5			433.2		
40–64	200.4			363.1		
≥ 65	562.2			853.7		
Time since arrival in Canada						
Less than 3 years	281.6	281.9	272.2–291.7	473.6	456.2	452.1–460.2
3–7 years	330.2	359.0	340.4–377.7	401.0	402.5	400.0–405.1
8 or more years	20.6	44.4	31.2–57.7	326.8	325.7	323.6–327.8
Immigrant admission category						
Temporary residents						
Worker	397.4	307.3	288.1–326.6			
Student	139.5	141.6	124.3–159.0			
Asylum claimant	137.8	158.8	153.3–164.3			
Permanent residents						
Economic				252.0	282.4	279.9–284.9
Family				560.2	476.2	473.1–479.2
Refugee				518.9	531.3	525.0–537.6
Sex						
Male	73.2	86.7	82.3–91.0	212.0	217.0	215.1–218.9
Female	425.9	313.2	308.4–317.9	527.1	512.3	510.1–514.4
Source world region						
USA	207.0	193.0	179.7–206.2	343.7	417.0	406.1–427.9
Caribbean/Central and South America	258.1	230.2	215.2–245.2	440.8	435.2	439.3–441.1
Western Europe	278.8	251.3	229.1–273.6	345.8	370.8	363.7–377.8
Eastern Europe	224.2	200.8	191.3–210.2	416.0	397.2	391.7–402.6
Sub-Saharan Africa	219.3	193.1	183.9–202.3	534.2	531.8	523.5–540.2
Southwest Asia with North Africa	151.1	144.3	133.0–155.6	385.5	397.4	392.6–402.1
South Asia	190.6	144.1	138.5–149.8	412.2	391.1	388.1–394.0
Southeast Asia	500.9	384.4	370.1–398.7	387.5	404.3	399.8–408.8
East Asia	167.8	127.5	118.5–136.4	244.3	253.9	251.4–256.5
Oceania	205.0^a^	188.8^a^	154.6–223.0	407.5	412.0	389.0–435.0

Source: 2014–2018 IMDB-DAD-OMHRS linked database, linked through the Longitudinal Immigration Statistical Environment (LISE), a secure linkage environment containing anonymous linkage keys of a subset of databases

^a^Use with caution due to high coefficient of variation

All-cause crude and ASHRs for TR and PR subgroups are summarized in Table 3. The TR subgroups had lower all-cause ASHRs compared to PR subgroups, similar to TRs and PRs overall (Table 2). However, those with an immigration status transition (e.g., TR-PR or PR-TR) had higher all-cause ASHRs compared to those who did not (e.g., TRs or PRs only). Further, differences in all-cause ASHR were observed between migration categories within the TR and PR subgroups. For example, students had higher all-cause ASHR in the TR-PR subgroup compared to TRs only. Like PRs overall, PR-TRs and PRs only in the economic class had the lowest ASHR, followed by the family and refugee classes. TRs only from Eastern Europe and sub-Saharan Africa had the highest all-cause ASHRs while TR-PRs from Southeast Asia and the USA had the highest. Meanwhile, PRs only from sub-Saharan Africa and Oceania had the highest all-cause ASHRs while PR-TRs from Southeast Asia and sub-Saharan Africa had the highest.

**Table 3:** All-cause age-standardized hospitalization rates (ASHR) for temporary and permanent resident subgroups, by selected characteristics (per 10,000 life years)

	Temporary residents (TRs)				Permanent residents (PRs)			
	Group A: TR only		Group B: TR who became PR		Group A: PR only		Group B: PR who were previously TR	
	ASHR	95% confidence interval	ASHR	95% confidence interval	ASHR	95% confidence interval	ASHR	95% confidence interval
Overall	117.8	109.9–125.8	232.9	228.6–237.2	361.6	360.0–363.3	444.2	439.9–448.5
Time since arrival in Canada								
Less than 3 years	104.3	97.2–111.4	376.7	361.1–392.3	463.7	459.0–468.5	491.9	479.0–504.9
3–7 years	169.7^a^	132.3–207.1	424.3	402.8–445.8	392.9	390.0–395.7	453.9	446.8–460.9
8 or more years	333.4^a^	263.2–403.5	42.4	26.3–58.5	312.3	310.1–314.5	402.1	395.1–409.1
Immigrant admission category								
Temporary residents								
Worker	98.0	91.5–104.4	381.2^a^	346.6–415.8				
Student	76.3	60.2–92.3	205.2	174.1–236.2				
Asylum claimant	300.0	274.9–325.2	149.0	143.2–154.8				
Permanent residents								
Economic					257.4	254.6–260.2	366.7	360.7–372.7
Family					483.6	480.2–487.0	465.6	545.8–476.3
Refugee					566.2	557.0–575.4	488.3	479.4–497.2
Sex								
Male	91.3	81.8–100.9	95.8	89.6–102.0	206.8	204.7–208.8	272.9	267.0–278.9
Female	158.0	144.6–171.5	382.5	376.5–388.5	488.6	486.2–491.0	611.7	605.5–617.9
Source world region								
USA	76.0	66.8–85.1	355.1	322.8–387.5	413.2	398.1–428.3	424.8	408.7–440.8
Caribbean/Central and South America	178.2^a^	141.9–214.4	260.0	242.2–277.8	412.8	405.7–419.9	480.1	468.3–491.9
Western Europe	110.0	90.5–129.5	309.6^a^	274.4–344.7	353.9	345.1–362.8	394.5	379.9–409.1
Eastern Europe	262.1^a^	197.1–327.0	213.2	202.4–224.0	379.3	373.3–385.3	470.5	456.7–484.3
Sub-Saharan Africa	228.0^a^	189.8–266.2	206.2	195.7–216.7	533.5	523.8–543.1	524.3	508.0–540.6
Southwest Asia with North Africa	181.6^a^	144.9–218.3	157.5	143.5–171.5	397.8	392.8–402.8	417.1	401.2–432.9
South Asia	200.2^a^	160.2–240.2	150.5	144.2–156.7	390.3	387.3–393.4	434.4	422.4–446.4
Southeast Asia	173.1^a^	134.6–211.6	401.2	386.1–416.4	358.4	353.5–363.3	544.3	524.3–564.3
East Asia	79.9	59.6–100.3	193.4	182.7–204.2	231.4	228.6–234.3	316.6	308.0–325.3
Oceania	92.7^a^	39.4–146.0	261.7^a^	215.8–307.5	442.2^a^	411.4–473.0	389.7^a^	348.2–431.3

Source: 2014–2018 IMDB-DAD-OMHRS linked database, linked through the Longitudinal Immigration Statistical Environment (LISE), a secure linkage environment containing anonymous linkage keys of a subset of databases

^a^Use with caution due to high coefficient of variation

### Cause-specific ASHR

Crude rates and ASHRs for cancer, injury, and mental health conditions among TRs and PRs are summarized in Table 4. Overall, TRs had lower crude rates and ASHRs for each health condition compared to PRs, similar to all-cause ASHR differences observed in TRs and PRs. Workers in the TR cohort had the highest ASHR for cancer and injury, while refugees in the PR cohort had the highest rates for each health condition. Notably, students in the TR cohort had the second highest ASHR for cancer but the lowest ASHR for injury. Asylum claimants in the TR cohort and refugees in the PR cohort had the highest ASHR for mental health conditions. Economic PRs had the lowest ASHRs for each health condition.

**Table 4:** Crude and age-standardized hospitalization rate (ASHR) for specific health conditions by temporary and permanent residents overall, by immigrant categories (per 10,000 life years)

	Crude rate	ASHR	95% confidence interval
Cancer			
Temporary residents	9.6	11.0	10.4–11.7
Worker	16.7	23.2	20.9–25.4
Student	2.6	8.2	4.7–11.7
Asylum claimant	5.0	4.7	4.1–5.4
Permanent residents	24.4	24.6	24.3–24.9
Economic	19.3	21.4	20.8–22.1
Family	32.6	28.2	27.5–28.8
Refugee	27.8	31.6	30.4–32.8
Injury			
Temporary residents	9.1	9.8	9.3–10.3
Worker	13.4	17.9	13.9–21.9
Student	4.5	5.1	3.3–7.0
Asylum claimant	6.2	7.7	6.8–8.6
Permanent residents	19.3	19.5	19.2–19.8
Economic	13.1	14.8	14.4–15.3
Family	26.5	22.6	22.0–23.2
Refugee	30.6	32.5	31.4–33.6
Mental health-related conditions (including self-inflicted injuries)			
Temporary residents	7.4	8.0	7.1–8.9
Worker	7.4	12.5^a^	6.8–18.2^a^
Student	5.4	5.5	3.7–7.2
Asylum claimant	9.7	13.6	10.6–16.6
Permanent residents	21.0	21.0	20.6–21.5
Economic	15.0	15.3	14.8–15.8
Family	23.6	24.7	23.7–25.8
Refugee	43.7	41.9	40.0–43.8

Source: 2014–2018 IMDB-DAD-OMHRS linked database, linked through the Longitudinal Immigration Statistical Environment (LISE), a secure linkage environment containing anonymous linkage keys of a subset of databases

^a^Use with caution due to high coefficient of variation

For TRs and PRs, the top three diseases for hospitalization under cancer, injury, and mental health conditions are presented in Supplementary Table 2. For cancer hospitalizations, more than a third (34%) of PRs were hospitalized for benign neoplasms, while almost 40% of TRs were hospitalized for digestive organ neoplasms. The distribution of injury-related hospitalizations was similar for TRs and PRs: a higher proportion of PRs (29%) were hospitalized for surgical and medical care complications compared to TRs (22%), while a higher proportion of TRs (19%) were hospitalized for injuries to the knee, lower leg, and neck compared to PRs (14%). For mental health conditions, a higher proportion of TRs (33%) were hospitalized for bipolar, depressive, and other mood disorders compared to PRs (27%).

## Discussion

This descriptive study compared all-cause crude and ASHRs between TRs and PRs, stratified by age, sex, world region of origin, migration category, and TR to PR transition status, and examined cause-specific ASHRs by migration category. Overall, the all-cause and cause-specific ASHRs were lower for TRs than for PRs. However, all-cause and cause-specific ASHRs varied across migration categories and among migrants with TR to PR status transitions. The all-cause ASHRs were also much higher for females than for males among both TRs and PRs, likely because pregnancy-related hospitalizations were included in the all-cause ASHR calculations.

Among PRs, the economic class had the lowest all-cause and cause-specific ASHRs. This observation is consistent with several previous studies that examined health outcomes among the PR immigrant categories studies (Morassaei et al., 2022; Ng et al., 2021, 2016a, 2016b; Woodgate et al., 2017). In contrast, refugees had the highest all-cause ASHR. Refugees often experience trauma and other negative health exposures before and during the immigration process that can result in poorer health outcomes (Gabriel et al., 2011). Further, there is more variation in education, language proficiency, and socioeconomic status among refugees and they may experience communication, health literacy, and health system barriers (Hynie, 2018; Saberpor, 2016; Woodgate et al., 2017). Refugees had the highest ASHR for mental health conditions, injury, and cancer, which is consistent with findings in previous studies (Grundy et al., 2023; Hynie, 2018; Uphoff et al., 2020). Refugees may have traumatic experiences that contribute to lasting mental health concerns such as post-traumatic stress disorder, depression, and anxiety. They also experience a myriad of post-migration stressors such as loss of income, inability to work in their qualified field after resettlement, and potential barriers to support post-settlement (James et al., 2019) that force them to take on dangerous and precarious employment. Several studies (Vahabi et al., 2016; Walker et al., 2022) found that cancer screening among refugees was the lowest as compared with other immigrant groups due to numerous barriers, which may contribute to late diagnoses. Further, certain refugee populations have a higher prevalence of colon and breast cancer (Vahabi et al., 2016; Waheed et al., 2022), which are compounded by screening challenges.

Interestingly, this study found that ASHRs among TRs were lower than among PRs overall and for all specific causes examined. There are multiple possible explanations for these observations. Research on the healthy immigrant effect has shown that recent arrivals in Canada are generally in good health, especially among 20–65 year olds, and are less likely to report poor health conditions (Lu & Ng, 2019; Vang et al., 2015). Further, temporary residents such as students and workers staying in Canada for more than 6 months must complete medical screening requirements and demonstrate good health for work and study (Immigration, Refugees and Citizenship Canada, 2002). Another hypothesis for lower observed ASHRs among TRs could be the salmon effect, whereby migrants return to their country of origin when they become severely ill rather than seek treatment in the host country. Past studies have observed the salmon effect among migrants, which may explain why TRs have lower ASHRs and appear to have better health compared to PRs, despite experiencing numerous health system and socioeconomic barriers (Namer & Razum, 2018; Turra & Elo, 2008). Temporary residents may also experience gaps in healthcare coverage upon arrival in Canada that prevent them from seeking health services as universal healthcare does not cover all TRs. Specific TR groups that do qualify may experience gaps and variations in coverage across provinces and territories (Garasia et al., 2023; Woodgate et al., 2017).

Among TRs, temporary foreign workers had the highest all-cause ASHR. Previous studies (Ahmed et al., 2016; Preibisch & Hennebry, 2011; Tsai & Ghahari, 2023) have shown that migrants in Canada experience cultural, socioeconomic, communication, health system, and health literacy barriers when accessing primary healthcare. Other studies (Ahmed et al., 2016; Kuile et al., 2007; Preibisch & Hennebry, 2011; Salami et al., 2019; Tsai & Ghahari, 2023) have highlighted that migrant workers experience poorer health in Canada due to their exposures to dangerous employment, long work schedules, and higher risk of infectious diseases. One study found that between 2001 and 2011, 4.6 per 1000 migrant workers in Ontario were medically repatriated to their home country (Orkin et al., 2014). Migrant workers may also increase their risk tolerance to protect their employment, but are less inclined to seek healthcare for fear of losing their work or immigration status (Preibisch & Hennebry, 2011). Further, workers had the highest ASHRs for cancer and injury, consistent with previous studies that showed an increased risk of work-related injury (e.g., falls, fractures, musculoskeletal disorders) and risk of cancer from both exposures to toxic substances and delayed access to cancer screening among migrant workers (Hargreaves et al., 2019; Hennebry et al., 2016; Hynie, 2018; Landry et al., 2021; Mills et al., 2009; Preibisch & Hennebry, 2011; Sterud et al., 2018). Migrant workers may also experience barriers to outpatient services and may not have adequate health insurance, which may cause delays to treatment until health conditions become severe and require hospitalization (Hennebry et al., 2016; Preibisch & Hennebry, 2011). One study (Saunders et al., 2018) showed that recent migrants were more likely to visit emergency departments (ED) as a first point of contact because of poorer access to outpatient healthcare services, and another study (Hynie et al., 2016) showed that temporary foreign workers are at higher risk of being without health insurance, which mirrored the increase in percentage of ED visits by those who are uninsured.

Among TRs, asylum claimants had the highest ASHR for mental health conditions. Asylum claimants are people who have been forced to leave their homes and likely have experienced trauma and distress during and long after their forced displacement that can lead to persistent mental health concerns after arrival in Canada (Blackmore et al., 2020; Uphoff et al., 2020). Other post-migration stressors such as family separation or loss, language barriers, poverty, stigma, loss of status, and difficulty accessing culturally appropriate healthcare are all common experiences that compound poor mental health (Li et al., 2016; Uphoff et al., 2020) and could explain the higher mental health hospitalization rates observed in this group. Asylum claimants may also remain TRs longer as they wait for their applications to process and thus may have accrued more follow-up time as TRs during which potential hospitalizations could occur.

This study divided TRs and PRs into four subgroups to examine the potential impact of TR to PR transitions on hospitalization rates in both cohorts. New immigration pathways focused on transitioning TRs to PRs were recently created and results of this study can inform the health outcomes of this group. Overall, TR-PRs and PR-TRs had higher all-cause ASHRs compared to TRs only or PRs only. Previous studies (Ahmed et al., 2016; Kuile et al., 2007; Morassaei et al., 2022; Ng et al., 2016a, 2016b) have illustrated the differences in healthcare access across different migrant groups, suggesting findings from the present study could be related to the impact of immigration status transitions on changes to healthcare-seeking behaviours and access. For example, as TRs transition to PRs, they may have greater access to health benefits with the status change. However, this study found further ASHR variations among TR and PR categories, with or without immigration status transitions. Asylum claimants who were TRs only had the highest ASHR, while workers who were TR-PRs had the highest ASHRs. Workers may continue to experience poorer health outcomes during and after transitioning from TR to PR, which can be a lengthy process (Nakache & Dixon-Perera, 2015). Further, people who transition from TR to PR and reach retirement age in Canada receive access to other income benefits such as pension entitlements (Koning & Banting, 2013) that may improve their overall socioeconomic ability to pay for uninsured healthcare services compared to those who remained as TRs. Several previous studies have observed that asylum claimants have higher health needs than other immigrant groups but experience significant barriers and gaps in healthcare access (Hwang, 2017; Ng et al., 2021; Saberpor, 2016).

### Strengths and limitations

This study leveraged the LISE and its IMDB-DAD-OMHRS linked database to compare all-cause and cause-specific TR and PR hospitalizations in Canada. The linked database included TRs and PRs from across Canada and allowed for population-level examination of hospitalization events (excluding Quebec) by different migration-related variables. In the LISE, TRs could be followed across multiple consecutive permits that allowed for comparison of hospitalization rates to PRs over the same period of time. A large proportion of the literature focuses on comparing PRs to Canadian-born health outcomes (Koning & Banting, 2013). In contrast, this study contributes to limited research examining and comparing differences across different TR and PR migrant categories.

There are also limitations to this study. The analysis only captured acute-care inpatient hospitalizations: outpatient hospitalizations (e.g., day surgery) and health system use outside of the hospital setting were not examined. The National Ambulatory Care Reporting System was considered for linkage with the DAD to capture outpatient hospitalizations, but this is challenging because different regions in Canada submit data to each database. Individuals generally present to the hospital when their health condition becomes severe, such that hospitalizations may not fully represent the burden of conditions treated on a primarily outpatient basis (Ng et al., 2016a, 2016b). In addition, the data are not fully representative of the Canadian migrant population as hospitalization data from Quebec are not available in the DAD or OMHRS and not included. However, the DAD is one of the most nationally representative databases and would capture the Canadian population outside of Quebec. Visitors were not included in the data as they typically stay in Canada for short-term periods, which may not be consecutive and would be difficult to track. Finally, this was a descriptive study examining the differences in ASHR between TRs and PRs. Future research may wish to take a multivariate approach to control for any confounding effects introduced by specific factors (e.g., country of origin or application type) and to isolate the factors driving the ASHR differences between TRs and PRs.

## Conclusion

This study found that all-cause hospitalization rates were lower among TRs than among PRs overall, and the highest hospitalization rates within each of these groups were among workers and refugees, respectively. Hospitalization rates overall were higher for those with TR to PR transitions than for those who remained TR only or PR only. Further analysis is needed to determine the specific factors driving these differences between TRs and PRs, in conjunction with other immigration health indicator data like key informant interviews and population health surveys similar to previous studies (Carlos & Wilson, 2018; Nakache & Dixon-Perera, 2015; Woodgate et al., 2017). The results of additional research combined with those of the current study could inform health policy and planning, expand on a limited literature comparing health outcomes of different migrant populations, highlight specific vulnerable migrant groups that may need additional support to reduce hospitalization, and improve continuity of care.

## Contributions to knowledge

What does this study add to existing knowledge?

- This study found that all-cause hospitalization rates overall are lower among temporary residents (TRs) than among permanent residents (PRs) in Canada.
- Those with a TR to PR status transition had higher all-cause hospitalization rates overall compared to those who remained TR only or PR only.
- Workers, asylum claimants, and refugees had the highest all-cause hospitalization rates and hospitalization rates for injury, cancer, and mental health conditions.

What are the key implications for public health interventions, practice, or policy?

- Results of this study suggest that workers, asylum claimants, and refugees have higher hospitalization rates. Better understanding of health outcomes for these specific migrant groups is important to improve their post-migration health.

## Supplementary Information

Below is the link to the electronic supplementary material.

Supplementary file1 (DOCX 23 KB)

## Data Availability

The Longitudinal Immigration Statistical Environment (LISE), which includes the Longitudinal Immigration Database (IMDB) linked to the Discharge Abstract Database (DAD) and the Ontario Mental Health Reporting System (OMHRS), is available through Statistics Canada’s Research Data Centres.

## Code availability

Available upon request to the corresponding author.

## References

[CR1] Ahmed, S., Shommu, N. S., Rumana, N., Barron, G. R. S., Wicklum, S., & Turin, T. C. (2016). Barriers to access of primary healthcare by immigrant populations in Canada: A literature review. *Journal of Immigrant and Minority Health,**18*(6), 1522–1540. 10.1007/s10903-015-0276-z 26364053 10.1007/s10903-015-0276-z

[CR2] Blackmore, R., Boyle, J. A., Fazel, M., Ranasinha, S., Gray, K. M., Fitzgerald, G., Misso, M., & Gibson-Helm, M. (2020). The prevalence of mental illness in refugees and asylum seekers: A systematic review and meta-analysis. *PLOS Medicine,**17*(9), e1003337. 10.1371/journal.pmed.1003337 32956381 10.1371/journal.pmed.1003337 PMC7505461

[CR3] Canadian Institute for Health Information. (2021). *Hospital Mental Health Database Metadata (HMHDB) | CIHI*. https://www.cihi.ca/en/hospital-mental-health-database-metadata-hmhdb. Accessed Aug 2023.

[CR4] Carlos, J. K., & Wilson, K. (2018). Migration among temporary foreign workers: Examining health and access to health care among Filipina live-in caregivers. *Social Science and Medicine,**209*, 117–124. 10.1016/j.socscimed.2018.05.045 29859389 10.1016/j.socscimed.2018.05.045

[CR5] Gabriel, P. S., Morgan-Jonker, C., Phung, C. M. W., Barrios, R., & Kaczorowski, J. (2011). Refugees and health care–The need for data: Understanding the health of government-assisted refugees in Canada through a prospective longitudinal cohort. *Canadian Journal of Public Health,**102*(4), 269–272. 10.1007/BF03404047 21913581 10.1007/BF03404047 PMC6973582

[CR6] Garasia, S., Bishop, V., Clayton, S., Pinnington, G., Arinze, C., & Jalil, E. (2023). Health outcomes, health services utilization, and costs consequences of medicare uninsurance among migrants in Canada: A systematic review. *BMC Health Services Research,**23*(1), 427. 10.1186/s12913-023-09417-4 37138351 10.1186/s12913-023-09417-4 PMC10154752

[CR7] Grundy, A., Ng, E., Rank, C., Quinlan, J., Giovinazzo, G., Viau, R., Ponka, D., & Garner, R. (2023). Mental health and neurocognitive disorder–related hospitalization rates in immigrants and Canadian-born population: A linkage study. *Canadian Journal of Public Health,**114*(4), 692–704. 10.17269/s41997-023-00740-1 36809592 10.17269/s41997-023-00740-1 PMC10348999

[CR8] Gushulak, B. D., Pottie, K., Roberts, J. H., Torres, S., & DesMeules, M. (2011). Migration and health in Canada: Health in the global village. *CMAJ,**183*(12), E952–E958. 10.1503/cmaj.090287 20584934 10.1503/cmaj.090287 PMC3168671

[CR9] Hargreaves, S., Rustage, K., Nellums, L. B., McAlpine, A., Pocock, N., Devakumar, D., Aldridge, R. W., Abubakar, I., Kristensen, K. L., Himmels, J. W., Friedland, J. S., & Zimmerman, C. (2019). Occupational health outcomes among international migrant workers: A systematic review and meta-analysis. *The Lancet Global Health,**7*(7), e872–e882. 10.1016/S2214-109X(19)30204-9 31122905 10.1016/S2214-109X(19)30204-9 PMC6565984

[CR10] Hennebry, J., McLaughlin, J., & Preibisch, K. (2016). Out of the loop: (In)access to health care for migrant workers in Canada. *Journal of International Migration and Integration,**17*(2), 521–538. 10.1007/s12134-015-0417-1

[CR11] Hwang, J. (2017). Public health response and health status of asylum seekers: A review of the international literature and implications for the Canadian context. *National Collaborating Centre for Infectious Diseases*. https://nccid.ca/publications/public-health-response-health-status-asylum-seekers-review-international-literature-implications-canadian-context/. Accessed Aug 2023.

[CR12] Hynie, M. (2018). The social determinants of refugee mental health in the post-migration context: A critical review. *The Canadian Journal of Psychiatry,**63*(5), 297–303. 10.1177/0706743717746666 29202665 10.1177/0706743717746666 PMC5912301

[CR13] Hynie, M., Ardern, C. I., & Robertson, A. (2016). Emergency room visits by uninsured child and adult residents in Ontario, Canada: What diagnoses, severity and visit disposition reveal about the impact of being uninsured. *Journal of Immigrant and Minority Health,**18*(5), 948–956. 10.1007/s10903-016-0351-0 26860406 10.1007/s10903-016-0351-0

[CR14] Immigration, Refugees and Citizenship Canada. (2002). *Medical exams for visitors, students and workers* [Service initiation]. https://www.canada.ca/en/immigration-refugees-citizenship/services/application/medical-police/medical-exams/requirements-temporary-residents.html. Accessed Aug 2023.

[CR15] Immigration, Refugees and Citizenship Canada. (2022). *2022 Annual Report to Parliament on Immigration*. https://www.canada.ca/en/immigration-refugees-citizenship/corporate/publications-manuals/annual-report-parliament-immigration-2022.html. Accessed Aug 2023.

[CR16] Immigration, Refugees and Citizenship Canada. (2023). *Canada welcomes historic number of newcomers in 2022* [News releases]. https://www.canada.ca/en/immigration-refugees-citizenship/news/2022/12/canada-welcomes-historic-number-of-newcomers-in-2022.html. Accessed Aug 2023.

[CR17] James, P., Iyer, A., & Webb, T. L. (2019). The impact of post-migration stressors on refugees’ emotional distress and health: A longitudinal analysis. *European Journal of Social Psychology,**49*(7), 1359–1367. 10.1002/ejsp.2589

[CR18] Koning, E. A., & Banting, K. G. (2013). Inequality below the surface: Reviewing immigrants’ access to and utilization of five Canadian welfare programs. *Canadian Public Policy,**39*(4), 581–601. 10.3138/CPP.39.4.581

[CR19] Kuile, S., Rousseau, C., Munoz, M., Nadeau, L., & Ouimet, M. (2007). The universality of the Canadian health care system in question: Barriers to services for immigrants and refugees. *International Journal of Migration, Health and Social Care,**3*(1), 15–26. 10.1108/17479894200700003

[CR20] Landry, V., Semsar-Kazerooni, K., Tjong, J., Alj, A., Darnley, A., Lipp, R., & Guberman, G. I. (2021). The systemized exploitation of temporary migrant agricultural workers in Canada: Exacerbation of health vulnerabilities during the COVID-19 pandemic and recommendations for the future. *Journal of Migration and Health,**3*, 100035. 10.1016/j.jmh.2021.100035 34405185 10.1016/j.jmh.2021.100035 PMC8352132

[CR21] Li, S. S. Y., Liddell, B. J., & Nickerson, A. (2016). The relationship between post-migration stress and psychological disorders in refugees and asylum seekers. *Current Psychiatry Reports,**18*(9), 82. 10.1007/s11920-016-0723-0 27436307 10.1007/s11920-016-0723-0

[CR22] Lu, C., & Ng, E. (2019). Healthy immigrant effect by immigrant category in Canada. *Health Reports,**30*(4), 3–11. 10.25318/82-003-x201900400001-eng 30994921 10.25318/82-003-x201900400001-eng

[CR23] Lu, H., Chen, J., Wang, W., Wu, L., Shen, X., Yuan, Z., & Yan, F. (2015). Efforts to reduce the disparity between permanent residents and temporary migrants: Stop TB experiences in Shanghai, China. *Tropical Medicine & International Health: TM & IH,**20*(8), 1033–1040. 10.1111/tmi.12512 25819348 10.1111/tmi.12512

[CR24] Mills, P. K., Dodge, J., & Yang, R. (2009). Cancer in migrant and seasonal hired farm workers. *Journal of Agromedicine,**14*(2), 185–191. 10.1080/10599240902824034 19437276 10.1080/10599240902824034

[CR25] Minister of Justice. (2001). *Immigration and Refugee Protection Act*. https://www.laws-lois.justice.gc.ca/eng/acts/I-2.5/FullText.html. Accessed Aug 2023.

[CR26] Morassaei, S., Irvin, E., Smith, P. M., Wilson, K., & Ghahari, S. (2022). The role of immigrant admission classes on the health and well-being of immigrants and refugees in Canada: A scoping review. *Journal of Immigrant and Minority Health,**24*(4), 1045–1060. 10.1007/s10903-022-01352-6 35303219 10.1007/s10903-022-01352-6

[CR27] Nakache, D., & Dixon-Perera, L. (2015). *Temporary or transitional? Migrant workers’ experiences with permanent residence in Canada* (SSRN Scholarly Paper 2732661). https://papers.ssrn.com/abstract=2732661. Accessed Aug 2023.

[CR28] Namer, Y., & Razum, O. (2018). Convergence theory and the salmon effect in migrant health. *In Oxford Research Encyclopedia of Global Public Health*. 10.1093/acrefore/9780190632366.013.17

[CR29] Ng, E., Quinlan, J., Giovinazzo, G., Grundy, A., Rank, C., Syoufi, M., Ponka, D., & Garner, R. (2021). All-cause acute care hospitalization rates of immigrants and the Canadian-born population: A linkage study. *Health Reports,**32*(9), 3–13. 10.25318/82-003-x202100900001-eng 34523869 10.25318/82-003-x202100900001-eng

[CR30] Ng, E., Sanmartin, C., Elien-Massenat, D., & Manuel, D. G. (2016a). Vaccine-preventable disease-related hospitalization among immigrants and refugees to Canada: Study of linked population-based databases. *Vaccine,**34*(37), 4437–4442. 10.1016/j.vaccine.2016.06.079 27452866 10.1016/j.vaccine.2016.06.079

[CR31] Ng, E., Sanmartin, C., & Manuel, D. G. (2016b). Acute care hospitalization, by immigrant category: Linking hospital data and the Immigrant Landing File in Canada. *Health Reports,**27*(8), 12–18. 27532621

[CR32] Orkin, A. M., Lay, M., McLaughlin, J., Schwandt, M., & Cole, D. (2014). Medical repatriation of migrant farm workers in Ontario: A descriptive analysis. *Canadian Medical Association Open Access Journal,**2*(3), E192–E198. 10.9778/cmajo.20140014 10.9778/cmajo.20140014 PMC4183168 25295239

[CR33] Preibisch, K., & Hennebry, J. (2011). Temporary migration, chronic effects: The health of international migrant workers in Canada. *CMAJ,**183*(9), 1033–1038. 10.1503/cmaj.090736 21502343 10.1503/cmaj.090736 PMC3114894

[CR34] Saberpor, T. (2016). Refugee and asylum seekers in Canada: Barriers to health care services. *Glendon Journal of International Studies*, *9*. https://gjis.journals.yorku.ca/index.php/gjis/article/view/40238. Accessed Aug 2023.

[CR35] Salami, B., Salma, J., & Hegadoren, K. (2019). Access and utilization of mental health services for immigrants and refugees: Perspectives of immigrant service providers. *International Journal of Mental Health Nursing,**28*(1), 152–161. 10.1111/inm.12512 29984880 10.1111/inm.12512

[CR36] Saunders, N. R., Gill, P. J., Holder, L., Vigod, S., Kurdyak, P., Gandhi, S., & Guttmann, A. (2018). Use of the emergency department as a first point of contact for mental health care by immigrant youth in Canada: A population-based study. *CMAJ,**190*(40), E1183–E1191. 10.1503/cmaj.180277 30301742 10.1503/cmaj.180277 PMC6175628

[CR37] Statistics Canada. (2022). *SDLE: overview. Social Data Linkage Environment (SDLE)*. https://www.statcan.gc.ca/en/sdle/overview. Accessed Aug 2023.

[CR38] Sterud, T., Tynes, T., Mehlum, I. S., Veiersted, K. B., Bergbom, B., Airila, A., Johansson, B., Brendler-Lindqvist, M., Hviid, K., & Flyvholm, M.-A. (2018). A systematic review of working conditions and occupational health among immigrants in Europe and Canada. *BMC Public Health,**18*, 770. 10.1186/s12889-018-5703-3 29925349 10.1186/s12889-018-5703-3 PMC6011510

[CR39] Tsai, P.-L., & Ghahari, S. (2023). Immigrants’ experience of health care access in Canada: A recent scoping review. *Journal of Immigrant and Minority Health,**25*(3), 712–727. 10.1007/s10903-023-01461-w 36870008 10.1007/s10903-023-01461-w

[CR40] Turra, C. M., & Elo, I. T. (2008). The impact of salmon bias on the Hispanic mortality advantage: New evidence from social security data. *Population Research and Policy Review,**27*(5), 515–530. 10.1007/s11113-008-9087-4 19122882 10.1007/s11113-008-9087-4 PMC2546603

[CR41] Uphoff, E., Robertson, L., Cabieses, B., Villalón, F. J., Purgato, M., Churchill, R., & Barbui, C. (2020). An overview of systematic reviews on mental health promotion, prevention, and treatment of common mental disorders for refugees, asylum seekers, and internally displaced persons. *The Cochrane Database of Systematic Reviews,**9*(9), CD013458. 10.1002/14651858.CD013458.pub2 32885850 10.1002/14651858.CD013458.pub2 PMC8572368

[CR42] Vahabi, M., Lofters, A., Kumar, M., & Glazier, R. H. (2016). Breast cancer screening disparities among immigrant women by world region of origin: A population-based study in Ontario, Canada. *Cancer Medicine,**5*(7), 1670–1686. 10.1002/cam4.700 27105926 10.1002/cam4.700 PMC4944895

[CR43] Vang, Z., Sigouin, J., Flenon, A., & Gagnon, A. (2015). The healthy immigrant effect in Canada: A systematic review. *Population Change and Lifecourse Strategic Knowledge Cluster Discussion Paper Series/ Un Réseau Stratégique de Connaissances Changements de Population et Parcours de Vie Document de Travail*, *3*(1). https://ir.lib.uwo.ca/pclc/vol3/iss1/4. Accessed Aug 2023.

[CR44] Waheed, A., McCloskey, A., Kennedy, F., Seraj, S. M., Khan, J., Nama, N., Johnson, O., Lo, P., Magee, H., Akbar, W., Ullah, A., & Cason, F. D. (2022). Colorectal cancer screening challenges in the recent Afghan refugee population: A comprehensive review article. *Cureus,**14*(2), e22400. 10.7759/cureus.22400 35345684 10.7759/cureus.22400 PMC8939286

[CR45] Walker, P. F., Settgast, A. M., & DeSilva, M. B. (2022). Cancer screening in refugees and immigrants: A global perspective. *The American Journal of Tropical Medicine and Hygiene,**106*(6), 1593–1600. 10.4269/ajtmh.21-0692 35533696 10.4269/ajtmh.21-0692 PMC9209943

[CR46] Wallar, L. E., & Rosella, L. C. (2020). Risk factors for avoidable hospitalizations in Canada using national linked data: A retrospective cohort study. *PLoS ONE,**15*(3), e0229465. 10.1371/journal.pone.0229465 32182242 10.1371/journal.pone.0229465 PMC7077875

[CR47] Wiedmeyer, M.-L., Goldenberg, S., Peterson, S., Wanigaratne, S., Machado, S., Tayyar, E., Braschel, M., Carrillo, R., Sierra-Heredia, C., Tuyisenge, G., & Lavergne, M. R. (2023). SARS-CoV-2 testing and COVID-19-related primary care use among people with citizenship, permanent residency, and temporary immigration status: An analysis of population-based administrative data in British Columbia. *Canadian Journal of Public Health,**114*(3), 389–403. 10.17269/s41997-023-00761-w 37014576 10.17269/s41997-023-00761-w PMC10072010

[CR48] Woodgate, R. L., Busolo, D. S., Crockett, M., Dean, R. A., Amaladas, M. R., & Plourde, P. J. (2017). A qualitative study on African immigrant and refugee families’ experiences of accessing primary health care services in Manitoba, Canada: It’s not easy! *International Journal for Equity in Health,**16*(1), 5. 10.1186/s12939-016-0510-x 28068998 10.1186/s12939-016-0510-x PMC5223444

