# Supplementary material for: All-cause and cause-specific hospitalization rates among temporary and permanent residents living in Canada: A linkage study
Source: Can J Public Health. 2025 Mar 18;116(5):698–709. doi: 10.17269/s41997-025-00996-9 (PMC12678690; doi:10.17269/s41997-025-00996-9)
Supplement: Supplementary file 1 — Supplementary file1 (DOCX 23 KB) [file 41997_2025_996_MOESM1_ESM.docx]

**Supplementary Table 1:** Hospitalization causes from the International Classification of Disease and Related Health Problems, Canada (ICD-10-CA).

| **Medical Conditions** | **ICD-10-CA Codes** |
| --- | --- |
| **Vaccine-preventable diseases (VPD)** |  |
| Tetanus  Diphtheria | A33  A36 |
| Pertussis (Whooping Cough) | A37 |
| Meningococcal infection | A39 |
| Sepsis due to Haemophilus Influenza | A41.3 |
| Acute Poliomyelitis | A80 |
| Varicella [chickenpox] | B01 |
| Zoster [herpes zoster] | B02 |
| Measles | B05 |
| Rubella | B06 |
| Mumps | B26 |
| Chronic viral hepatitis B | B18.0, B18.1, B19.1 |
| Pneumococcal infections | G00.1 |
| Other infectious diseases | A00 – B99 |
| **Cancer** | C00 – D49 |
| **Injury** | S10 – T88 |
| **Mental health conditions** | F10 to F99, X60 to X84 |
| **Tuberculosis** | A15 to A19, B90 |
|  |  |

Note: X codes used for self-inflicted injuries under mental health conditions do not appear as the most responsible diagnosis. Instead injury S or T codes are listed as the most responsible diagnosis. Hence, a certain proportion of hospitalizations would be classified as both mental health and injury-related hospitalizations.

**Supplementary Table 2:** Percentage distribution of top 3 diseases within each specific cause requiring hospitalization among temporary and permanent residents

| **Medical Conditions** | **ICD-10 codes** | **Temporary Residents** | **Permanent Residents** |
| --- | --- | --- | --- |
| **Cancer** |  |  |  |
| Benign neoplasms | D10-D36 | 13% | 34% |
| Neoplasm of digestive organs | C15-C26 | 37% | 19% |
| Neoplasms of eye, brain and other parts of the central nervous system | C69-C72 | 9% | 9% |
| **Injury** |  |  |  |
| Complications of surgical and medical care | T80-T88 | 22% | 29% |
| Injuries to the knee and lower leg | S80-S89 | 19% | 14% |
| Injuries to the neck | S10-S19 | 13% | 12% |
|  |  |  |  |
| **Mental health-related conditions** |  |  |  |
| Schizophrenia spectrum and other psychotic disorders | F20; F21; F22-F25; F28- F29 | 26% | 29% |
| Bipolar and related disorders; depressive disorders; other mood disorders | F30; F31; F34.0 | 33% | 27% |
| All others in ICD-10 Chapter 5 not included in the CIHI listing | F50-F59, F70-F99 | 19% | 16% |
| **Vaccine-preventable diseases (VPD)** |  |  |  |
| Chronic viral hepatitis B | B18.0, B18.1, B19.1 | 65% | 61% |
| Zoster [herpes zoster] | B02 | 25% | 25% |
| Varicella [chickenpox] | B01 | N/A | 7% |
|  |  |  |  |
| **Other infectious diseases** |  |  |  |
| Other sepsis | A41 | 24% | 32% |
| Infectious gastroenteritis and colitis | A09 | 18% | 16% |
| Bacterial infection of unspecified site | A49 | 7% | 9% |
